# Crystal structure of pyridinium tetra­iso­thio­cyanato­dipyridine­chromium(III) pyridine monosolvate

**DOI:** 10.1107/S2056989019014488

**Published:** 2019-11-15

**Authors:** Tristan Neumann, Inke Jess, Christian Näther

**Affiliations:** a Institut für Anorganische Chemie, Christian-Albrechts-Universität Kiel, Max-Eyth Strasse 2, D-24118 Kiel, Germany

**Keywords:** crystal structure,discrete complex, chromium(III), pyridinium, hydrogen bonding

## Abstract

In the crystal structure of the title compound, (C_5_H_6_N)[Cr(NCS)_4_(C_5_H_5_N)_2_]·C_5_H_5_N, the Cr^III^ ions are octa­hedrally coordinated by four *N*-bonding thio­cyanate anions and two pyridine ligands into discrete negatively charged complexes, with the Cr^III^ ion, as well as the two pyridine ligands, located on crystallographic mirror planes. The mean planes of the two pyridine ligands are rotated with respect to each other by 90°. Charge balance is achieved by one protonated pyridine mol­ecule that is hydrogen bonded to one additional pyridine solvent mol­ecule, with both located on crystallographic mirror planes and again rotated by exactly 90°. The pyridinium H atom was refined as disordered between both pyridine N atoms in a 70:30 ratio, leading to a linear N—H⋯N hydrogen bond. In the crystal, discrete complexes are linked by weak C—H⋯S hydrogen bonds into chains that are connected by additional C—H⋯S hydrogen bonding *via* the pyridinium cations and solvent mol­ecules into layers and finally into a three-dimensional network.

## Abstract 2

The crystal structure of the title compound consists of discrete negatively charged [Cr(NCS)_4_(pyridine)_2_]^−^ complexes that are charge balanced by pyridinium cations and contains additional pyridine solvent mol­ecules that are linked by weak C—H⋯S hydrogen bonding into a three-dimensional network.

## Chemical context   

Coordination compounds with paramagnetic transition metals are of great inter­est because of their diverse magnetic properties (Cirera *et al.*, 2009 ▸; Giannopoulos *et al.*, 2014 ▸; Glaser, 2011 ▸; Yuan *et al.*, 2007 ▸). Those in which the metal cations are linked by small-sized ligands that can mediate magnetic exchange are of special importance because co-operative magnetic phenomena can be expected. Prominent examples for this class of ligands are azides, oxalates and cyanides (Wang *et al.*, 2005 ▸, 2008 ▸; Zhang *et al.*, 2012 ▸; Manson *et al.*, 2005 ▸; Ding *et al.*, 2012 ▸). In this context also, thio­cyanate ligands are useful because they show a large variety of coordination modes, with the μ-1,3-bridging mode as the most important (Jochim *et al.*, 2018 ▸; Mautner *et al.*, 2016 ▸, 2017 ▸; Shurdha *et al.*, 2013 ▸; Mekuimemba *et al.*, 2018 ▸; Wöhlert *et al.*, 2014*a* ▸; Werner *et al.*, 2015 ▸). It is noted that these compounds are frequently difficult to prepare because terminal *N*-coordination is usually preferred for 3*d* metal cations. Nevertheless, in recent years, an increasing number of bridging compounds have been reported, which might be traced back to the fact that several of them were prepared by thermal decomposition of precursors that contain terminal anionic ligands (Näther *et al.*, 2013 ▸). In this context, we and others have reported on several new thio­cyanate coordination polymers based on transition-metal thio­cyanates, in which the metal cations are linked by bridging anionic ligands into chains (Rams *et al.*, 2017 ▸; Baran *et al.*, 2019 ▸; Wöhlert *et al.*, 2013 ▸, 2014*b* ▸; Mautner *et al.*, 2018 ▸). Most of these compounds contain Mn^II^, Fe^II^, Co^II^, Ni^II^ or Cu^II^ cations, whereas no bridging compounds are reported with chromium. There is only one compound in which alternating Cr^III^ and K^+^ cations are bridged by μ-1,3-coordinating thio­cyanate anions into chains in which each cation is octa­hedrally sourrounded by two bridging thio­cyanate anions and four pyridine ligands (Kitanovski *et al.*, 2007 ▸). Therefore, we decided to investigated if similar compounds are available with chromium. Hence, CrCl_2_ was reacted with NH_4_NCS to prepare Cr(NCS)_2_ *in situ*, which is similar to the procedure we frequently use for the synthesis of thio­cyanate coordination polymers with other metal cations. Initially, pyridine was selected as the N-donor ligand, because most of our compounds are based on pyridine derivatives as co-ligands. In this reaction, crystals were obtained that were identified by single-crystal X-ray diffraction. This proved that a discrete cationic complex had formed.
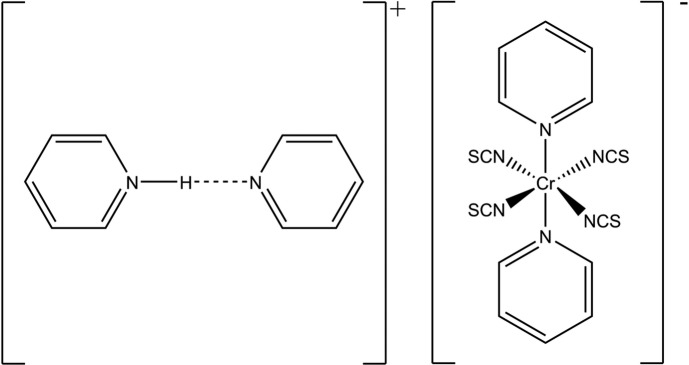


## Structural commentary   

**Figure 1:**
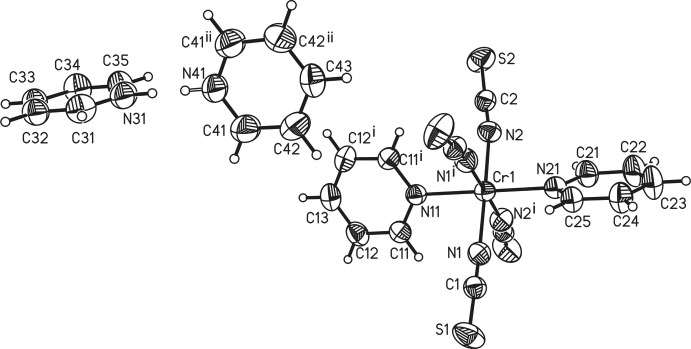
The mol­ecular structure of the title compound with the atom labelling and displacement ellipsoids drawn at the 50% probability level. The pyridinium N-bound H atom is disordered over two sets of sites.

**Figure 2:**
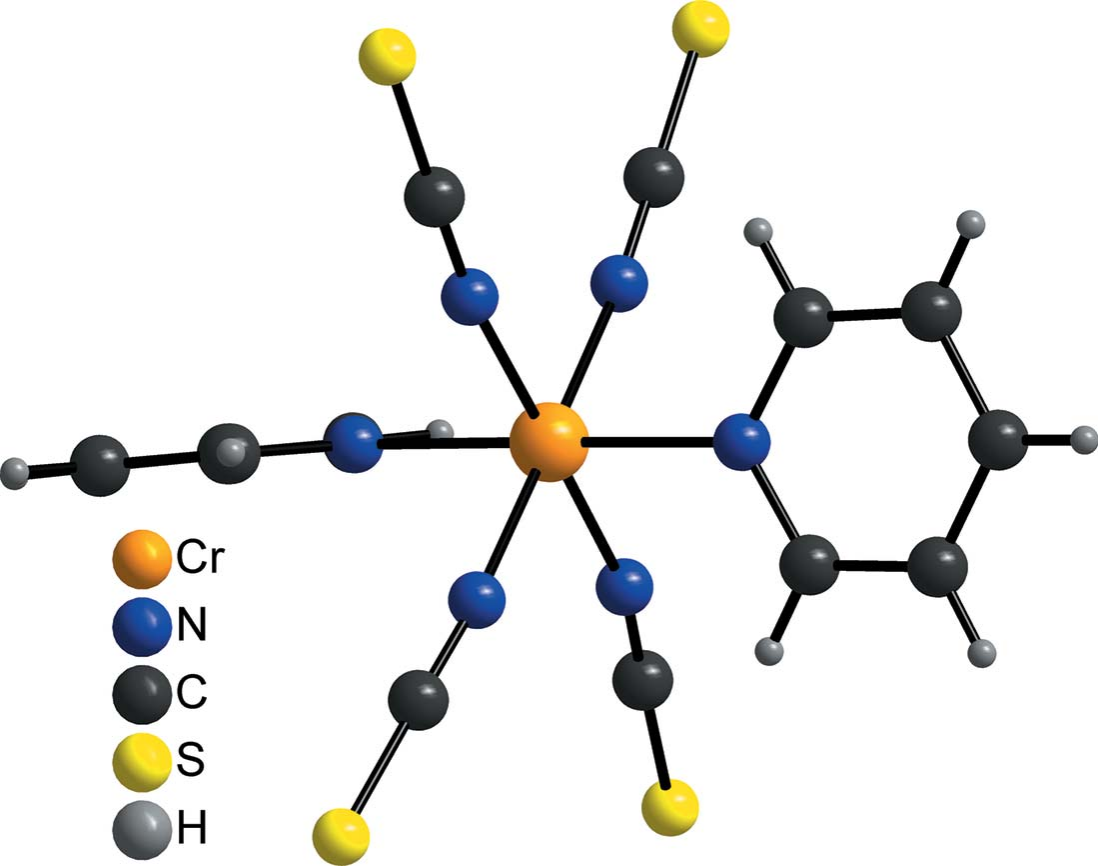
View of the coordination sphere of the Cr^III^ ion.

**Figure 3:**
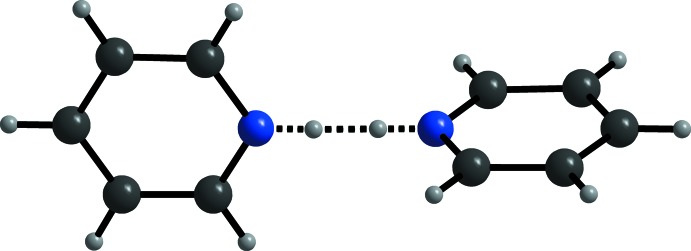
View of the pyridinium–pyridine dimer, with N–H⋯N hydrogen bonding shown as dashed lines. The pyridinium N-bound H atom is disordered over two sets of sites.

The asymmetric unit of the title compound consists of one half of the cation, namely a Cr^III^ ion, two pyridine ligands which lie on a crystallographic mirror plane and two iso­thio­cyanate anions that occupy general positions, as well as one pyridinium cation and a pyridine mol­ecule that are also located on a crystallographic mirror plane (Fig. 1 ▸). The Cr^III^ ion is sixfold coordinated by four *N*-bonding iso­thio­cyanate anions and two pyridine ligands, within a slightly distorted octa­hedral geometry (Figs. 1 ▸ and 2 ▸). The Cr—N bond lengths (Table 1 ▸) to the pyridine ligands (N11 and N21) are slightly longer than that to the iso­thio­cyanate anions (N1 and N2). The distortion of the octa­hedron is also obvious from the mean quadratic elongation of 1.0015 and the octa­hedral angle variance of 0.9447° calculated according to Robinson *et al.* (1971 ▸). The four iso­thio­cyanate anions are located in the basal plane of the octa­hedron, whereas the pyridine ligands are in apical positions with the pyridine ring planes rotated by 90° (Fig. 2 ▸). Charge balance is achieved by a pyridinium cation that is hydrogen bonded to a pyridine solvent mol­ecule *via* N—H⋯N hydrogen bonding, forming pyridinium–pyridine dimers (Fig. 3 ▸). Several models were tested, but in the final refinement, a split model was used, in which the pyridinium H atom is disordered over two sites in a ratio of 70:30. Presumably, because of sterical reasons, the pyridine-ring planes are perpendicular to each other (Fig. 3 ▸).

## Supra­molecular features   

**Figure 4:**
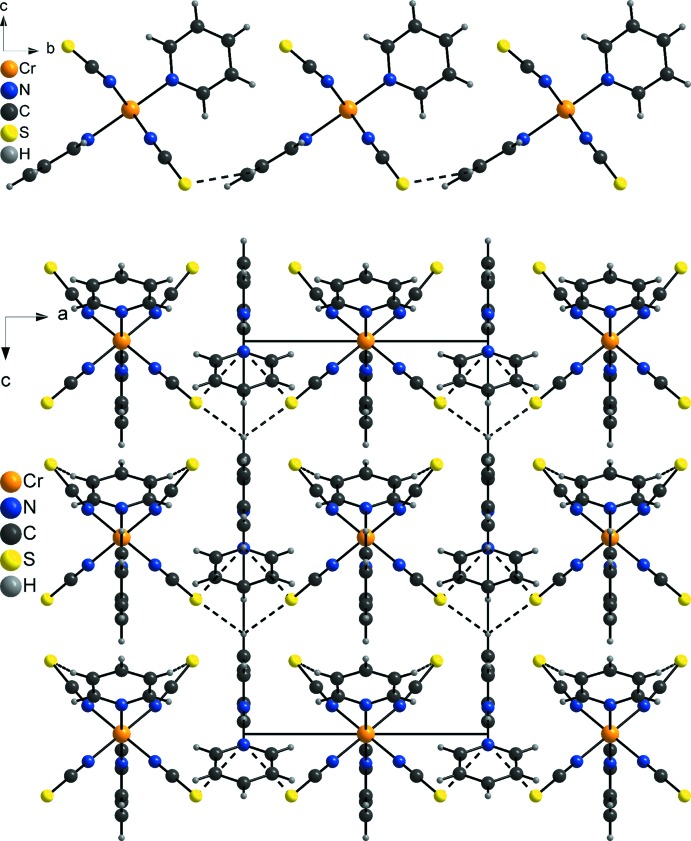
View of a chain (top) and the crystal structure of the title compound viewed along the crystallographic *b*-axis and with the inter­molecular hydrogen bonding shown as dashed lines.

In the crystal, discrete complexes and pyridinium cations are arranged in alternating layers parallel to the *bc* plane (Fig. 4 ▸, bottom). The discrete complexes are linked by pairs of C—H⋯S hydrogen bonds between the thio­cyanate S atoms of one complex and two H atoms of one of the pyridine ligands of a neighbouring complex into chains, that elongate along the crystallographic *b* axis (Fig. 4 ▸, top). The bond lengths and angles of these hydrogen bonds show that this is only a very weak inter­action (Table 2 ▸). These chains are further linked by additional very weak C—H⋯S inter­actions between the thio­cyanate S atoms that are not involved in chain formation and one H atom of the pyridinium–pyridine dimers (Fig. 4 ▸, bottom, and Table 2 ▸). Finally, further C—H⋯S inter­actions link all building blocks into a three-dimensional network. It is noted that both the discrete complexes, as well as the pyridinium–pyridine dimers, point in the same direction along the crystallographic *c* axis, clearly showing the presence of a polar structure (Fig. 4 ▸, bottom).

## Database survey   

There are four structures published in the CSD (Version 5.4, Update 1, February 2019; Groom *et al.*, 2016 ▸) that consist of chromium(II) and thio­cyanate anions. In all of them, the Cr^II^ cations are square-planar coordinated by two iso­thio­cyanate anions and two co-ligands (Jubb *et al.*, 1989 ▸, 1991 ▸; Shurdha *et al.*, 2012 ▸, 2013 ▸). With chromium(III) there are two structures in which the cations are octa­hedrally coordinated by only two terminal iso­thio­cyanate anions and by two 4,4′-dimethyl-2,2′-pyridine ligands and the positive charge is neutralized by iodide or triiodide anions (Walter & Elliott, 2001 ▸). In most of the reported structures with Cr^III^, the cations are surrounded by four or six iso­thio­cyanate anions and the positive charges are neutralized by protonated solvent or ligand mol­ecules. There is also one discrete complex with pyridine as co-ligand, in which the Cr^III^ cations are coordinated by three iso­thio­cyanate anions and three pyridine ligands (Malecki, 2016 ▸). A similar structure is also known with 4-methyl­pyridine (Kitanovski *et al.*, 2007 ▸). Finally, there is one structure reported that is comparable to that of the title compound with 4-methyl­pyridine, in which the Cr^III^ cations are coordinated by two 4-methyl­pyridine ligands and four *N*-terminal thio­cyanate anions. Charge balance is achieved by one 4-methyl­pyridinium cation that is hydrogen bonded to one 4-methyl­pyridine solvent mol­ecule (Young *et al.*, 2011 ▸). In contrast to the title compound, the N—H distances are not symmetrical (N—H = 1.16 Å and N⋯H = 1.5 Å), but the N—H⋯N hydrogen-bond distance is comparable (2.686 Å) to that in the title compound (2.684 Å).

## Synthesis and crystallization   

CrCl_2_ (0.5 mmol, 66.5 mg) was reacted with NH_4_NCS (1.0 mmol, 76.1 mg) in 2.0 ml pyridine. The precipitate was filtered off and the filtrate was stored at room temperature. After a few days, crystals of the title compound suitable for single-crystal x-ray diffraction were obtained.

## Refinement   

Crystal data, data collection and structure refinement details are summarized in Table 3 ▸. The H atoms were positioned with idealized geometry and refined with *U*_iso_(H) = 1.2*U*_eq_(C) using a riding model. The pyridinium H atom was located in a difference map and was initially freely refined. In this case, it is located nearly in the middle between both pyridine N atoms, leading to very long N—H bonds of 1.32 (16) and 1.35 (16) Å, an N—H⋯N angle close to linearity and a relatively large isotropic displacement parameter. For such N—H⋯N hydrogen bonds, both symmetric and asymmetric hydrogen bonds were determined by neutron diffraction, but the symmetric bonds are usually observed at shorter N⋯N distances (Rozière *et al.*, 1980 ▸, 1982 ▸). Therefore, the pyridinium H atom was placed at an ideal distance and the displacement parameter was refined. In this case, there is a strong indication that the H atom is disordered and therefore a split model was used with the site-occupation factor for each H atom in a ratio of 70:30, which leads to similar isotropic displacement parameters that are lower than that obtained by the refinement of a symmetrical hydrogen bond. In the final refinement, both H atoms were placed in ideal positions and were refined with *U*_iso_(H) = 1.2*U*_eq_(N) using a riding model.

## Supplementary Material

Crystal structure: contains datablock(s) I, global. DOI: 10.1107/S2056989019014488/lh5933sup1.cif

Structure factors: contains datablock(s) I. DOI: 10.1107/S2056989019014488/lh5933Isup2.hkl

CCDC references: 1962530, 1962530

Additional supporting information:  crystallographic information; 3D view; checkCIF report

## Figures and Tables

**Table 1:** Selected geometric parameters (Å, °)

Cr1—N2^i^	1.980 (5)	Cr1—N1^i^	1.991 (5)
Cr1—N2	1.980 (5)	Cr1—N21	2.080 (6)
Cr1—N1	1.991 (5)	Cr1—N11	2.102 (6)
			
N2^i^—Cr1—N2	88.4 (3)	N1—Cr1—N21	90.39 (18)
N2^i^—Cr1—N1	91.33 (18)	N1^i^—Cr1—N21	90.40 (18)
N2—Cr1—N1	178.9 (2)	N2^i^—Cr1—N11	88.98 (18)
N2^i^—Cr1—N1^i^	178.9 (2)	N2—Cr1—N11	88.98 (18)
N2—Cr1—N1^i^	91.33 (18)	N1—Cr1—N11	90.00 (18)
N1—Cr1—N1^i^	88.9 (3)	N1^i^—Cr1—N11	90.00 (18)
N2^i^—Cr1—N21	90.62 (18)	N21—Cr1—N11	179.4 (3)
N2—Cr1—N21	90.62 (18)		

Symmetry code: (i) 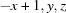.

**Table 2:** Hydrogen-bond geometry (Å, °)

*D*—H⋯*A*	*D*—H	H⋯*A*	*D*⋯*A*	*D*—H⋯*A*
C12—H12⋯S1^ii^	0.93	2.93	3.647 (6)	135
C32—H32⋯S2^iii^	0.93	3.01	3.719 (8)	134
C32—H32⋯S2^iv^	0.93	3.01	3.719 (8)	134
C35—H35⋯S2^v^	0.93	2.90	3.494 (7)	123
C35—H35⋯S2^vi^	0.93	2.90	3.494 (7)	123
N31—H31*A*⋯N41	0.86	1.82	2.684 (11)	179
N41—H41*A*⋯N31	0.86	1.82	2.684 (11)	180

Symmetry codes: (ii) 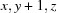; (iii) 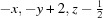; (iv) 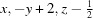; (v) 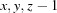; (vi) 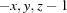.

**Table 3:** Experimental details

Crystal data	
Chemical formula	(C_5_H_6_N)[Cr(NCS)_4_(C_5_H_5_N)_2_]·C_5_H_5_N
*M*_r_	601.73
Crystal system, space group	Orthorhombic, *P* *m* *c*2_1_
Temperature (K)	293
*a*, *b*, *c* (Å)	10.1068 (5), 8.8168 (6), 16.2628 (9)
*V* (Å^3^)	1449.17 (15)
*Z*	2
Radiation type	Mo *K*α
μ (mm^−1^)	0.71
Crystal size (mm)	0.12 × 0.07 × 0.02

Data collection	
Diffractometer	Stoe IPDS1
Absorption correction	Numerical (*X-SHAPE* and *X-RED32*; Stoe & Cie, 2008 ▸)
*T*_min_, *T*_max_	0.786, 0.966
No. of measured, independent and observed [*I* > 2σ(*I*)] reflections	12253, 3153, 2452
*R*_int_	0.062
(sin θ/λ)_max_ (Å^−1^)	0.639

Refinement	
*R*[*F* ^2^ > 2σ(*F* ^2^)], *wR*(*F* ^2^), *S*	0.043, 0.106, 1.02
No. of reflections	3153
No. of parameters	193
No. of restraints	1
H-atom treatment	H-atom parameters constrained
Δρ_max_, Δρ_min_ (e Å^−3^)	0.30, −0.42
Absolute structure	Flack *x* determined using 925 quotients [(*I* ^+^)−(*I* ^−^)]/[(*I* ^+^)+(*I* ^−^)] (Parsons *et al.*, 2013 ▸)
Absolute structure parameter	−0.027 (19)

Computer programs: *X-AREA* (Stoe & Cie, 2008 ▸), *SHELXS97* (Sheldrick, 2008 ▸), *SHELXL2014* (Sheldrick, 2015 ▸), *XP* in *SHELXTL* (Sheldrick, 2008 ▸), *DIAMOND* (Brandenburg, 1999 ▸) and *publCIF* (Westrip, 2010 ▸).

## supplementary crystallographic information

### Crystal data

**Table:**

(C_5_H_6_N)[Cr(NCS)_4_(C_5_H_5_N)_2_]·C_5_H_5_N	*D*_x_ = 1.379 Mg m^−^^3^
*M**_r_* = 601.73	Mo *K*α radiation, λ = 0.71073 Å
Orthorhombic, *P**m**c*2_1_	Cell parameters from 12253 reflections
*a* = 10.1068 (5) Å	θ = 2.6–28.1°
*b* = 8.8168 (6) Å	µ = 0.71 mm^−^^1^
*c* = 16.2628 (9) Å	*T* = 293 K
*V* = 1449.17 (15) Å^3^	Plate, green
*Z* = 2	0.12 × 0.07 × 0.02 mm
*F*(000) = 618	

### Data collection

**Table:**

Stoe IPDS1 diffractometer	2452 reflections with *I* > 2σ(*I*)
Phi scans	*R*_int_ = 0.062
Absorption correction: numerical (X-SHAPE and X-RED32; Stoe & Cie, 2008)	θ_max_ = 27.0°, θ_min_ = 2.6°
*T*_min_ = 0.786, *T*_max_ = 0.966	*h* = −11→12
12253 measured reflections	*k* = −11→11
3153 independent reflections	*l* = −20→20

### Refinement

**Table:**

Refinement on *F*^2^	Hydrogen site location: inferred from neighbouring sites
Least-squares matrix: full	H-atom parameters constrained
*R*[*F*^2^ > 2σ(*F*^2^)] = 0.043	*w* = 1/[σ^2^(*F*_o_^2^) + (0.0503*P*)^2^ + 0.4808*P*] where *P* = (*F*_o_^2^ + 2*F*_c_^2^)/3
*wR*(*F*^2^) = 0.106	(Δ/σ)_max_ < 0.001
*S* = 1.02	Δρ_max_ = 0.30 e Å^−^^3^
3153 reflections	Δρ_min_ = −0.42 e Å^−^^3^
193 parameters	Absolute structure: Flack *x* determined using 925 quotients [(I+)-(I-)]/[(I+)+(I-)] (Parsons *et al.*, 2013)
1 restraint	Absolute structure parameter: −0.027 (19)

### Special details

**Table:**

Geometry. All esds (except the esd in the dihedral angle between two l.s. planes) are estimated using the full covariance matrix. The cell esds are taken into account individually in the estimation of esds in distances, angles and torsion angles; correlations between esds in cell parameters are only used when they are defined by crystal symmetry. An approximate (isotropic) treatment of cell esds is used for estimating esds involving l.s. planes.

### Fractional atomic coordinates and isotropic or equivalent isotropic displacement parameters (Å^2^)

**Table:**

	*x*	*y*	*z*	*U*_iso_*/*U*_eq_	Occ. (<1)
Cr1	0.500000	0.64497 (13)	0.99944 (7)	0.0350 (3)	
N1	0.6380 (5)	0.5526 (5)	0.9278 (3)	0.0456 (11)	
C1	0.7030 (6)	0.4870 (6)	0.8803 (3)	0.0409 (12)	
S1	0.7901 (2)	0.39515 (18)	0.81396 (11)	0.0671 (5)	
N2	0.3635 (5)	0.7405 (5)	1.0697 (3)	0.0453 (11)	
C2	0.2924 (6)	0.8219 (6)	1.1063 (3)	0.0410 (12)	
S2	0.1955 (2)	0.9312 (2)	1.15735 (11)	0.0689 (5)	
N11	0.500000	0.8403 (7)	0.9254 (4)	0.0378 (13)	
C11	0.6137 (6)	0.9059 (6)	0.9028 (3)	0.0446 (13)	
H11	0.693081	0.859637	0.917262	0.053*	
C12	0.6175 (7)	1.0412 (6)	0.8584 (4)	0.0522 (14)	
H12	0.697858	1.084674	0.843515	0.063*	
C13	0.500000	1.1087 (10)	0.8372 (5)	0.054 (2)	
H13	0.499999	1.200033	0.808600	0.065*	
N21	0.500000	0.4529 (7)	1.0738 (4)	0.0380 (14)	
C21	0.500000	0.4633 (10)	1.1565 (5)	0.051 (2)	
H21	0.500000	0.558953	1.180637	0.061*	
C22	0.500000	0.3371 (11)	1.2067 (6)	0.065 (3)	
H22	0.500000	0.348073	1.263584	0.078*	
C23	0.500000	0.1953 (12)	1.1719 (6)	0.068 (3)	
H23	0.500000	0.109435	1.205088	0.082*	
C24	0.500000	0.1805 (10)	1.0868 (6)	0.059 (2)	
H24	0.500000	0.085445	1.061946	0.070*	
C25	0.500000	0.3110 (9)	1.0405 (5)	0.0441 (18)	
H25	0.500000	0.301981	0.983543	0.053*	
N31	0.000000	1.0017 (9)	0.4322 (5)	0.061 (2)	
H31A	0.000000	0.920955	0.461881	0.073*	0.3
C31	0.000000	0.9933 (10)	0.3502 (6)	0.057 (2)	
H31	0.000000	0.898380	0.325264	0.068*	
C32	0.000000	1.1428 (12)	0.4678 (6)	0.062 (2)	
H32	0.000000	1.150849	0.524812	0.075*	
C33	0.000000	1.2701 (11)	0.4217 (6)	0.060 (2)	
H33	0.000000	1.364655	0.447130	0.072*	
C34	0.000000	1.2604 (10)	0.3374 (5)	0.051 (2)	
H34	0.000000	1.347438	0.305068	0.061*	
C35	0.000000	1.1195 (10)	0.3021 (5)	0.051 (2)	
H35	0.000000	1.109984	0.245176	0.062*	
N41	0.000000	0.7526 (8)	0.5271 (5)	0.0557 (19)	
H41A	0.000000	0.832178	0.496530	0.067*	0.7
C41	0.1138 (8)	0.6919 (8)	0.5505 (5)	0.0686 (19)	
H41	0.192859	0.735531	0.533295	0.082*	
C42	0.1165 (8)	0.5636 (8)	0.6004 (5)	0.071 (2)	
H42	0.196943	0.522486	0.617040	0.085*	
C43	0.000000	0.4985 (10)	0.6248 (5)	0.057 (2)	
H43	0.000001	0.411685	0.657268	0.069*	

### Atomic displacement parameters (Å^2^)

**Table:**

	*U*^11^	*U*^22^	*U*^33^	*U*^12^	*U*^13^	*U*^23^
Cr1	0.0346 (6)	0.0369 (5)	0.0333 (5)	0.000	0.000	−0.0032 (5)
N1	0.048 (3)	0.049 (3)	0.039 (2)	0.001 (2)	0.000 (2)	0.001 (2)
C1	0.040 (3)	0.037 (3)	0.045 (3)	−0.001 (2)	0.006 (3)	−0.001 (2)
S1	0.0761 (13)	0.0503 (8)	0.0749 (11)	−0.0015 (8)	0.0372 (9)	−0.0093 (8)
N2	0.045 (3)	0.047 (3)	0.044 (3)	0.002 (2)	0.006 (2)	−0.003 (2)
C2	0.040 (3)	0.045 (3)	0.038 (3)	0.001 (2)	−0.003 (2)	0.001 (2)
S2	0.0684 (12)	0.0846 (12)	0.0537 (9)	0.0326 (9)	0.0046 (9)	−0.0143 (8)
N11	0.036 (4)	0.040 (3)	0.037 (3)	0.000	0.000	−0.001 (3)
C11	0.041 (3)	0.046 (3)	0.047 (3)	−0.003 (2)	0.001 (2)	0.004 (2)
C12	0.059 (4)	0.045 (3)	0.052 (3)	−0.007 (3)	0.010 (3)	0.005 (3)
C13	0.071 (6)	0.045 (4)	0.046 (5)	0.000	0.000	0.006 (4)
N21	0.040 (4)	0.035 (3)	0.039 (3)	0.000	0.000	−0.004 (2)
C21	0.064 (6)	0.054 (5)	0.035 (4)	0.000	0.000	−0.001 (3)
C22	0.090 (8)	0.064 (6)	0.042 (4)	0.000	0.000	0.007 (4)
C23	0.096 (8)	0.059 (6)	0.050 (5)	0.000	0.000	0.022 (5)
C24	0.074 (7)	0.047 (5)	0.055 (5)	0.000	0.000	0.007 (4)
C25	0.051 (5)	0.042 (4)	0.039 (4)	0.000	0.000	0.001 (3)
N31	0.051 (5)	0.055 (4)	0.075 (5)	0.000	0.000	0.020 (4)
C31	0.059 (6)	0.045 (4)	0.066 (6)	0.000	0.000	−0.005 (4)
C32	0.066 (6)	0.076 (6)	0.045 (4)	0.000	0.000	0.003 (5)
C33	0.073 (7)	0.051 (5)	0.055 (5)	0.000	0.000	−0.010 (4)
C34	0.054 (5)	0.053 (5)	0.045 (4)	0.000	0.000	−0.001 (4)
C35	0.052 (5)	0.059 (5)	0.043 (4)	0.000	0.000	−0.009 (4)
N41	0.054 (5)	0.050 (4)	0.063 (5)	0.000	0.000	0.014 (3)
C41	0.051 (4)	0.064 (4)	0.091 (5)	0.000 (3)	0.004 (4)	0.017 (4)
C42	0.056 (5)	0.071 (4)	0.086 (5)	0.016 (3)	−0.002 (4)	0.018 (4)
C43	0.079 (7)	0.045 (4)	0.047 (5)	0.000	0.000	0.009 (4)

### Geometric parameters (Å, º)

**Table:**

Cr1—N2^i^	1.980 (5)	C23—H23	0.9300
Cr1—N2	1.980 (5)	C24—C25	1.375 (11)
Cr1—N1	1.991 (5)	C24—H24	0.9300
Cr1—N1^i^	1.991 (5)	C25—H25	0.9300
Cr1—N21	2.080 (6)	N31—C31	1.336 (13)
Cr1—N11	2.102 (6)	N31—C32	1.372 (13)
N1—C1	1.168 (7)	N31—H31A	0.8600
C1—S1	1.610 (6)	C31—C35	1.360 (13)
N2—C2	1.177 (7)	C31—H31	0.9300
C2—S2	1.605 (6)	C32—C33	1.350 (13)
N11—C11	1.338 (7)	C32—H32	0.9300
N11—C11^i^	1.338 (7)	C33—C34	1.375 (12)
C11—C12	1.395 (7)	C33—H33	0.9300
C11—H11	0.9300	C34—C35	1.368 (11)
C12—C13	1.373 (8)	C34—H34	0.9300
C12—H12	0.9300	C35—H35	0.9300
C13—H13	0.9300	N41—C41	1.324 (8)
N21—C21	1.349 (10)	N41—C41^ii^	1.324 (8)
N21—C25	1.363 (10)	N41—H41A	0.8600
C21—C22	1.379 (12)	C41—C42	1.393 (9)
C21—H21	0.9300	C41—H41	0.9300
C22—C23	1.373 (15)	C42—C43	1.369 (9)
C22—H22	0.9300	C42—H42	0.9300
C23—C24	1.390 (13)	C43—H43	0.9300
			
N2^i^—Cr1—N2	88.4 (3)	C22—C23—C24	119.8 (9)
N2^i^—Cr1—N1	91.33 (18)	C22—C23—H23	120.1
N2—Cr1—N1	178.9 (2)	C24—C23—H23	120.1
N2^i^—Cr1—N1^i^	178.9 (2)	C25—C24—C23	117.8 (9)
N2—Cr1—N1^i^	91.33 (18)	C25—C24—H24	121.1
N1—Cr1—N1^i^	88.9 (3)	C23—C24—H24	121.1
N2^i^—Cr1—N21	90.62 (18)	N21—C25—C24	123.5 (7)
N2—Cr1—N21	90.62 (18)	N21—C25—H25	118.3
N1—Cr1—N21	90.39 (18)	C24—C25—H25	118.3
N1^i^—Cr1—N21	90.40 (18)	C31—N31—C32	118.1 (8)
N2^i^—Cr1—N11	88.98 (18)	C31—N31—H31A	120.9
N2—Cr1—N11	88.98 (18)	C32—N31—H31A	120.9
N1—Cr1—N11	90.00 (18)	N31—C31—C35	121.9 (8)
N1^i^—Cr1—N11	90.00 (18)	N31—C31—H31	119.0
N21—Cr1—N11	179.4 (3)	C35—C31—H31	119.0
C1—N1—Cr1	169.7 (5)	C33—C32—N31	121.4 (9)
N1—C1—S1	178.9 (6)	C33—C32—H32	119.3
C2—N2—Cr1	167.6 (5)	N31—C32—H32	119.3
N2—C2—S2	179.1 (5)	C32—C33—C34	120.1 (9)
C11—N11—C11^i^	118.4 (7)	C32—C33—H33	119.9
C11—N11—Cr1	120.8 (3)	C34—C33—H33	119.9
C11^i^—N11—Cr1	120.7 (3)	C35—C34—C33	118.3 (8)
N11—C11—C12	122.4 (6)	C35—C34—H34	120.8
N11—C11—H11	118.8	C33—C34—H34	120.8
C12—C11—H11	118.8	C31—C35—C34	120.1 (8)
C13—C12—C11	118.5 (6)	C31—C35—H35	119.9
C13—C12—H12	120.8	C34—C35—H35	119.9
C11—C12—H12	120.8	C41—N41—C41^ii^	120.6 (8)
C12^i^—C13—C12	119.8 (8)	C41—N41—H41A	119.7
C12^i^—C13—H13	120.1	C41^ii^—N41—H41A	119.7
C12—C13—H13	120.1	N41—C41—C42	120.9 (7)
C21—N21—C25	117.3 (7)	N41—C41—H41	119.6
C21—N21—Cr1	121.6 (5)	C42—C41—H41	119.6
C25—N21—Cr1	121.1 (5)	C43—C42—C41	119.5 (7)
N21—C21—C22	122.4 (8)	C43—C42—H42	120.3
N21—C21—H21	118.8	C41—C42—H42	120.3
C22—C21—H21	118.8	C42—C43—C42^ii^	118.7 (8)
C23—C22—C21	119.4 (8)	C42—C43—H43	120.6
C23—C22—H22	120.3	C42^ii^—C43—H43	120.6
C21—C22—H22	120.3		

Symmetry codes: (i) −*x*+1, *y*, *z*; (ii) −*x*, *y*, *z*.

### Hydrogen-bond geometry (Å, º)

**Table:**

*D*—H···*A*	*D*—H	H···*A*	*D*···*A*	*D*—H···*A*
C12—H12···S1^iii^	0.93	2.93	3.647 (6)	135
C32—H32···S2^iv^	0.93	3.01	3.719 (8)	134
C32—H32···S2^v^	0.93	3.01	3.719 (8)	134
C35—H35···S2^vi^	0.93	2.90	3.494 (7)	123
C35—H35···S2^vii^	0.93	2.90	3.494 (7)	123
N31—H31*A*···N41	0.86	1.82	2.684 (11)	179
N41—H41*A*···N31	0.86	1.82	2.684 (11)	180

Symmetry codes: (iii) *x*, *y*+1, *z*; (iv) −*x*, −*y*+2, *z*−1/2; (v) *x*, −*y*+2, *z*−1/2; (vi) *x*, *y*, *z*−1; (vii) −*x*, *y*, *z*−1.

## References

[bb1] Baran, S., Hoser, A., Rams, M., Ostrovsky, S., Neumann, T., Näther, C. & Tomkowicz, Z. (2019). *J. Phys. Chem. Solids*, **130**, 290–297.

[bb2] Brandenburg, K. (1999). *DIAMOND*. Crystal Impact GbR, Bonn, Germany.

[bb3] Cirera, J., Ruiz, E., Alvarez, S., Neese, F. & Kortus, J. (2009). *Chem. Eur. J.* **15**, 4078–4087. 10.1002/chem.200801608 19248077

[bb4] Ding, M., Wang, B., Wang, Z., Zhang, J., Fuhr, O., Fenske, D. & Gao, S. (2012). *Chem. Eur. J.* **18**, 915–924. 10.1002/chem.201101912 22170388

[bb5] Giannopoulos, D. P., Thuijs, A., Wernsdorfer, W., Pilkington, M., Christou, G. & Stamatatos, T. C. (2014). *Chem. Commun.* **50**, 779–781. 10.1039/c3cc47094f 24281143

[bb6] Glaser, T. (2011). *Chem. Commun.* **47**, 116–130. 10.1039/c0cc02259d 20862425

[bb7] Groom, C. R., Bruno, I. J., Lightfoot, M. P. & Ward, S. C. (2016). *Acta Cryst.* B**72**, 171–179. 10.1107/S2052520616003954 PMC4822653 27048719

[bb8] Jochim, A., Rams, M., Neumann, T., Wellm, C., Reinsch, H., Wójtowicz, G. M. & Näther, C. (2018). *Eur. J. Inorg. Chem.* **2018**, 4779–4789.

[bb9] Jubb, J., Larkworthy, L. F., Leonard, G. A., Povey, D. C. & Tucker, B. J. (1989). *J. Chem. Soc. Dalton Trans.* pp. 1631–1633.

[bb10] Jubb, J., Larkworthy, L. F., Oliver, L. F., Povey, D. C. & Smith, G. W. (1991). *J. Chem. Soc. Dalton Trans.* pp. 2045–2050.

[bb11] Kitanovski, N., Golobič, A. & Čeh, B. (2007). *Croat. Chem. Acta*, **80**, 127–134.

[bb12] Malecki, J. G. (2016). *CSD Communication*, CCDC 767462. CCDC, Cambridge, England.

[bb13] Manson, J. L., Lancaster, T., Chapon, L. C., Blundell, S. J., Schlueter, J. A., Brooks, M. L., Pratt, F. L., Nygren, C. L. & Qualls, J. S. (2005). *Inorg. Chem.* **44**, 989–995. 10.1021/ic048723x 15859278

[bb14] Mautner, F. A., Berger, C., Fischer, R. C. & Massoud, S. S. (2016). *Inorg. Chim. Acta*, **439**, 69–76.

[bb15] Mautner, F. A., Fischer, R. C., Rashmawi, L. G., Louka, F. R. & Massoud, S. S. (2017). *Polyhedron*, **124**, 237–242.

[bb16] Mautner, F. A., Traber, M., Fischer, R. C., Torvisco, A., Reichmann, K., Speed, S., Vicente, R. & Massoud, S. S. (2018). *Polyhedron*, **154**, 436–442.

[bb17] Mekuimemba, C. D., Conan, F., Mota, A. J., Palacios, M. A., Colacio, E. & Triki, S. (2018). *Inorg. Chem.* **57**, 2184–2192. 10.1021/acs.inorgchem.7b03082 29420016

[bb18] Näther, C., Wöhlert, S., Boeckmann, J., Wriedt, M. & Jess, I. (2013). *Z. Anorg. Allg. Chem.* **639**, 2696–2714.

[bb19] Parsons, S., Flack, H. D. & Wagner, T. (2013). *Acta Cryst.* B**69**, 249–259. 10.1107/S2052519213010014 PMC3661305 23719469

[bb20] Rams, M., Böhme, M., Kataev, V., Krupskaya, Y., Büchner, B., Plass, W., Neumann, T., Tomkowicz, Z. & Näther, C. (2017). *Phys. Chem. Chem. Phys.* **19**, 24534–24544. 10.1039/c7cp04189f 28852749

[bb21] Robinson, K., Gibbs, G. V. & Ribbe, P. H. (1971). *Science*, **172**, 567–570. 10.1126/science.172.3983.567 17802221

[bb22] Rozière, J., Belin, C. & Lehman, M. S. (1982). *J. Chem. Soc. Chem. Commun.* pp. 388–389.

[bb23] Rozière, J., Williams, J. M., Grech, E., Malarski, Z. & Sobcyzk, L. (1980). *J. Chem. Phys.* **72**, 6117–6122.

[bb24] Sheldrick, G. M. (2008). *Acta Cryst.* A**64**, 112–122. 10.1107/S0108767307043930 18156677

[bb25] Sheldrick, G. M. (2015). *Acta Cryst.* C**71**, 3–8.

[bb26] Shurdha, E., Lapidus, S. H., Stephens, P. W., Moore, C. E., Rheingold, A. L. & Miller, J. S. (2012). *Inorg. Chem.* **51**, 9655–9665. 10.1021/ic300804y 22928927

[bb27] Shurdha, E., Moore, C. E., Rheingold, A. L., Lapidus, S. H., Stephens, P. W., Arif, A. M. & Miller, J. S. (2013). *Inorg. Chem.* **52**, 10583–10594. 10.1021/ic401558f 23981238

[bb28] Stoe & Cie (2008). *X-AREA*, *X-RED32* and *X-SHAPE*. Stoe & Cie, Darmstadt, Germany.

[bb29] Walter, B. J. & Elliott, C. M. (2001). *Inorg. Chem.* **40**, 5924–5927. 10.1021/ic0103291 11681906

[bb30] Wang, X.-Y., Wang, L., Wang, Z.-M., Su, G. & Gao, S. (2005). *Chem. Mater.* **17**, 6369–6380.

[bb31] Wang, X.-Y., Wang, Z.-M. & Gao, S. (2008). *Chem. Commun.* pp. 281–294. 10.1039/b708122g 18399189

[bb32] Werner, J., Runčevski, T., Dinnebier, R., Ebbinghaus, S. G., Suckert, S. & Näther, C. (2015). *Eur. J. Inorg. Chem.* **2015**, 3236–3245.

[bb33] Westrip, S. P. (2010). *J. Appl. Cryst.* **43**, 920–925.

[bb34] Wöhlert, S., Fic, T., Tomkowicz, Z., Ebbinghaus, S. G., Rams, M., Haase, W. & Näther, C. (2013). *Inorg. Chem.* **52**, 12947–12957. 10.1021/ic4012235 24171470

[bb35] Wöhlert, S., Runčevski, T., Dinnebier, R. E., Ebbinghaus, S. G. & Näther, C. (2014*a*). *Cryst. Growth Des.* **14**, 1902–1913.

[bb36] Wöhlert, S., Tomkowicz, Z., Rams, M., Ebbinghaus, S. G., Fink, L., Schmidt, M. U. & Näther, C. (2014*b*). *Inorg. Chem.* **53**, 8298–8310. 10.1021/ic500572p 25080077

[bb37] Young, J. L., Harris, J. D., Benjamin, J. A., Fitch, J. E., Nogales, D. F., Walker, J. R., Frost, B. J., Thurber, A. & Punnoose, A. (2011). *Inorg. Chim. Acta*, **377**, 14–19.

[bb38] Yuan, M., Zhao, F., Zhang, W., Pan, F., Wang, Z.-M. & Gao, S. (2007). *Chem. Eur. J.* **13**, 2937–2952. 10.1002/chem.200601174 17171734

[bb39] Zhang, X.-M., Wang, Y.-Q., Li, X.-B. & Gao, E.-Q. (2012). *Dalton Trans.* **41**, 2026–2033. 10.1039/c1dt11692d 22183258

